# The Effect of Observers’ Mood on the Local Processing of Emotional Faces: Evidence from Short-Lived and Prolonged Mood States

**DOI:** 10.5709/acp-0167-5

**Published:** 2015-03-31

**Authors:** Setareh Mokhtari, Heather Buttle

**Affiliations:** School of Psychology, Massey University, Auckland, New Zealand

**Keywords:** attention, emotion, face perception, global-local processing, prolonged vs. short-lived mood

## Abstract

We examined the effect of induced mood, varying in valence and longevity, on local processing of emotional faces. It was found that negative facial expression conveyed by the global level of the face interferes with efficient processing of the local features. The results also showed that the duration of involvement with a mood influenced the local processing. We observed that attending to the local level of faces is not different in short-lived happy and sad mood states. However, as the mood state is experienced for a longer period, local processing was impaired in happy mood compared to sad mood. Taken together, we concluded that both facial expressions and affective states influence processing of the local parts of faces. Moreover, we suggest that mediating factors like the duration of involvement with the mood play a role in the interrelation between mood, attention, and perception.

## Introduction

The existing literature broadly accepts that affective states are able to influence the way we process our complex environment. Many theories have been developed to explain the relationship between mood and processing strategy in compound stimuli. A common theme in some of these theories is that there is a pre-set and fixed link between a particular mood valence and visual perception strategies (e.g., Fredrickson, 1998, 2004; Fredrickson & Branigan, 2005; Gasper, 2004; Gasper & Clore, 2002); while others consider the relationship between mood and perception to be more flexible (e.g., Baumann & Kuhl, 2005).

The *levels-of-focus hypothesis* (Gasper, 2004; Gasper & Clore, 2002), for instance, suggests that each happy or sad mood promotes a specific level of processing; happy mood is associated with global level processing and sad mood is linked with processing of the local details. This hypothesis refers to the *affect-as-information approach* which assumes that, the appropriateness of the accessible mental strategies is evaluated by the mood valence (Clore, Gasper, & Garvin, 2001; Clore, Wyer, et al., 2001). Happy mood signals that the situation is harmless and accessible mental resources are trustworthy. Since global level perception is assumed to be the most accessible strategy in processing the compound stimuli (Kimchi, 1992; Navon, 1977), happy mood assures the person that they can rely on the global processing to deal with the situation. However, sad mood warns the observer that the situation is problematic and the available mental resources are not helpful. Following this stop signal, using the most accessible strategies (i.e., global processing) is suppressed and the probability of adopting the local level processing strategy increases (Gasper, 2004; Gasper & Clore, 2002).

Along the lines of the levels-of-focus hypothesis, the *broaden-and-build theory* also implies the pre-set effect of happy mood on expanding the scope of attention over the global feature of the situation (Fredrickson & Branigan, 2005; Hicks & King, 2007). This theory postulates that attending to the global level enables a person to discover the whole environment and build new mental resources which have extensive adaptive values (Fredrickson, 1998, 2004). Therefore happy mood, by broadening the attentional resources over the global level, has survival advantages.

While the above mentioned hypotheses have linked a particular perceptual strategy with a specific mood valence, other theories have adopted a more flexible approach (Baumann & Kuhl, 2005; Tan, Jones, & Watson, 2009). For example, Bauman and Kuhl discussed that different mood valences are not necessarily associated with any processing strategy preference, but provide various levels of flexibility for the cognitive mechanism to switch from one strategy to another. Happy mood equips the observer with a cognitive flexibility to overcome the dominant perceptual strategies and switch to the most satisfactory strategy, if required (Baumann & Kuhl, 2005). In comparison, sad mood reduces the flexibility of perceptual mechanisms to switch from an accessible perceptual strategy to another. For example, if the observer is required to detect a target embedded in the local level of a shape, adopting a local processing strategy is more informative and helpful in fulfilling the task requirement. In this situation, the perceptual system should have a flexibility to suppress the prominent global processing strategy and switch to a non-prominent (i.e., local) processing strategy. Contrary to the level-of-focus hypothesis, Bauman and Kuhl’s results showed that happy mood, compared to sad mood, facilitates the ability of the perceptual system to overcome the global perceptual strategy and adopt the local perceptual strategy (see also Tan et al., 2009).

For many years, researchers’ main focus has centred on investigating how different task situations can influence the relation of mood and perception; but, the quality of experienced moods and its plausible effects have not received the same amount of attention. Studies of the impact of mood on perception mostly rely on laboratory-based mood induction procedures (MIPs) in which, by the use of one or more techniques participants’ mood is manipulated in the laboratory (Gerrards-Hesse, Spies, & Hesse, 1994; Martin, 1990; Westermann, Spies, Stahl, & Hesse, 1996). Despite the extensive application of different MIPs, these procedures are noticeably different in the quality of induced mood (e.g., Westermann et al., 1996).

Critical observation of the available literature reveals that usually the main concern of researchers was to assure that the desired mood valence (i.e., happy or sad mood) was significantly induced in research participants. This means, apart from the state of pleasantness or unpleasantness, other characteristics of the induced mood are usually ignored by researchers. However, we suggest that studying the relationship between mood and perception will be inadequate if other characteristics of the experienced mood are not taken into account. One factor that might play a role in connection between mood and perception is the duration of involvement with a certain mood. For example, Gilboa, Roberts, and Gotlib (1997) noticed that people who often experienced sad mood, as opposed to those who were temporarily induced into sad mood in an experimental setting, showed more negative bias in memory recall. This evidence points to the necessity of studying the effect of mood longevity as an important factor in guiding cognitive resources.

In this context, the current study aimed to investigate the influence of happy and sad induced mood, varying in exposure duration, on local level processing. We aimed to design a target detection task in which participants have to actively suppress the global level information and adopt a local processing strategy. To fulfil this aim, the human face was the best stimulus for our task because it has a compound structure and because previous studies have extensively shown that global processing is the prominent strategy in face perception, and processing of the facial features without prior attention to the global face gestalt is impossible (e.g., Mermelstein, Banks, & Prinzmetal, 1979; Navon, 1977; Suzuki & Cavanagh, 1995). In one study, Eastwood, Smilek, and Merikle (2003) showed that efficiency of switching attention from the global level of a face to its elemental features is influenced by the emotional meaning of the overall face gestalt. In their experiments participants were requested to count a target shape embedded in emotional faces. The experimental displays consisted of four schematic faces expressing either positive or negative emotion. The results showed that attending to the elemental parts was affected by the emotional meaning conveyed by the global level of faces: Allocation of attention to the facial features of the negative faces was significantly slower than with the positive faces. The authors suggested that negative facial expressions involuntarily capture and hold attention and interfere with the effective switching of attention to the elemental parts. Faster attending to the elements of positive faces cannot be due to the efficient processing of the global level of positive faces since no difference between positive and neutral faces was observed (Eastwood et al., 2003). Eastwood et al. suggested that the global level of the face interferes with early processing of local details even if it was irrelevant to the task requirements.

## The current study

There were three main aims in this study: First, we intended to compare the interference of positive and negative facial expressions, conveyed by the global level of faces, on efficient attentional switching to the elemental parts. Second, we aimed to investigate in a local processing task whether happy or sad mood facilitates attentional switching from the global face gestalt to the local facial features. Third, we considered whether duration of exposure with a happy or sad mood induction technique can manipulate the ease of attending to the facial features. In order to follow these aims, short-lived or prolonged happy or sad mood was induced in participants. We used a target detection task in which counting the number of target shapes (i.e., curves and straight lines) embedded in the schematic positive or negative faces was required. Faster reaction times would indicate the successful overcoming of the prominent global processing and efficient allocation of attention to the elemental parts of the faces.

If the allocation of attention to the elemental parts was directed only by the emotional meaning of the global level of faces, we hypothesised that the local processing would not be influenced by observers’ happy or sad mood. In this case, as suggested by Eastwood et al. (2003) faster reaction time in counting the elemental parts of positive rather than negative expressive faces was expected. However, if different mood states affect local processing of emotional faces, happy or sad mood would be able to either facilitate or inhibit switching of attention from the global face gestalt to the local details. Regarding the levels-of-focus hypothesis, in comparison with happy mood, faster reaction time to the facial details would be expected in sad mood. Whereas, based on the flexibility hypothesis, a faster reaction time in attending to the facial details was predicted in happy rather than sad mood. This research also looked for plausible interactions between emotional content of the global level of faces and affective states of observers in processing of facial features. If happy or sad mood results in mood-congruent stimuli biases—as proposed by Bower (1981)—we hypothesised that participants in happy mood prefer positive information, expressed by the global level of the faces. This attentional bias would decelerate ignoring the global level information and attending to the local features of positive faces. While the cognitive bias towards negative faces in participants with sad mood would interfere with disengaging attention from the overall negative expressive face gestalt and adopt a local processing strategy.

## Method

### Participants

Forty-five volunteers (26 women, 19 men) ranging in age from 18 to 40 years, fluent in English, with normal or corrected-to-normal vision volunteered to participate in the experiment in exchange for NZ $15. Participants were tested individually. The research design and procedure was approved by Massey University Human Ethical Committee: Northern (MUHEC: N) no.08/066R.

### Materials and design

#### Mood Induction Procedures

In order to induce a short-lived happy or sad mood, a combination of music mood induction procedure and autobiographic memory recall was used before completing the task. To induce a prolonged mood state, the same music pieces were played continuously during the course of the task.

Mozart’s *Eine Kleine Nachtmusik* was used to induce happy mood and Barber’s *Adagio for strings* was used to induce sad mood in participants. Participants were asked to remember one personal memory which was congruent with the emotion of the music. The selected music pieces have been shown to be effective in inducing intended moods by previous research (e.g., Chepenik, Cornew, & Farah, 2007; Nguyen & Scharff, 2003; Niedenthal & Setterlund, 1994; Sousou, 1997; Storbeck & Clore, 2005). The music was played through PC speakers with medium volume in the lab.

#### Visual task materials

**Figure 1.**
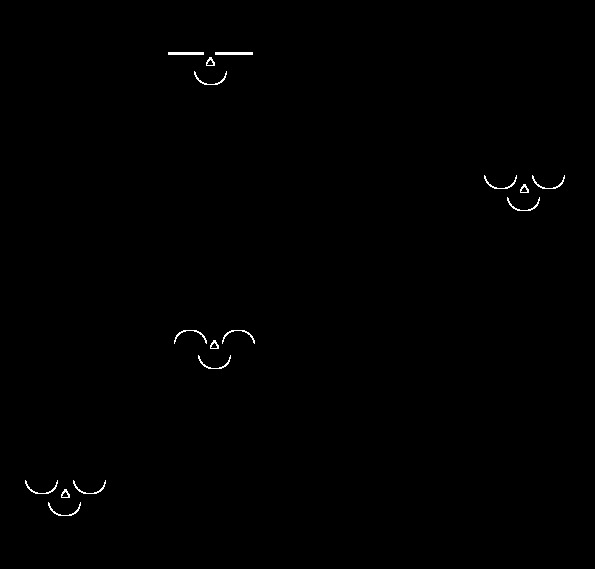
A sample of a positive face display presented in an imaginary 4 × 4 matrix (Figures are not drawn to scale).

The experimental task was presented on a PC with a 17 inch monitor. Each of the visual face displays contained four schematic faces expressing either positive or negative expressions, which randomly occupied four potential 2.5 × 2.5 cm cells in an imaginary 4 × 4 matrix. Each of the positive or negative faces consisted of suitable organization of three geometric forms (upward and downward bent curves, and straight lines) for eyes and mouths, with a size of 0.86ş width and 0.38ş height of visual angle. The direction of the mouth curvature was upward in positive expressions and downward in negative expressions. One triangle acting as a nose was located under the eyes and over the mouth in order to make each schematic figure similar to a real face. Each face subtended a visual angle of approximately 2.1° × 1.2°. The faces were presented in white colour on a black background (Figure 1). The stimulus design was adopted from Eastwood et al. (2003), and was also used in our previous study (Mokhtari & Buttle, 2011). The research task was programmed in C++ at the School of Psychology, Massey University. The experiment had a 2 (Face Display: Positive facial expression, Negative facial expression; within-subject factor) × 2 (Mood: Happy, Sad; between-subjects factor) × 2 (Mood Induction Technique: Short-lived, Prolonged; between-subjects factor) design. The main dependent variables were participants’ reaction times and accuracy of responses.

### Procedure

Participants were informed about the task and their rights upon arrival, with written consent obtained from each participant. Then, they were randomly assigned to one of the four mood induction groups: Short-lived Happy, Short-lived Sad, Prolonged Happy, and Prolonged Sad. Each person scored his/her own mood before and after mood induction on a 7- point scale, in which 1, 2, and 3 represented different levels of sad mood, 5, 6, and 7 represented different levels of happy mood and 4 showed a neutral mood state. Participants in both short-lived and prolonged mood manipulation groups were instructed to listen to the mood-appropriate piece of music and recall one of their mood-appropriate memories for 3 min and a half. The music continued to play during the rest of the experimental session for those participants who were allocated to the prolonged mood induction groups.

The first mood rating score provided a pre-test baseline. Increases in mood score after happy mood induction and decreases in mood score after sad mood induction were expected. Then participants were seated facing the computer screen with a viewing distance of 60 cm. The instructions were presented on the screen, but participants were allowed to ask questions if they encountered any difficulties. The task was to count the number of downward or upward bent curves or straight lines embedded in a face display. To inform the participants which of the mentioned shapes had to be counted, in each trial the target shape (e.g., a upward bent curve) flashed on the screen for 1,000 ms before being replaced with one of the positive or negative face displays. Participants were asked to count the targets as fast and accurately as possible. After they finished counting, participants pressed the spacebar as fast as possible and then recorded the number of targets by using the number pad. The responses were recorded by the computer. In total 162 trials were presented to each participant in a random order. The number of trials for positive and negative face displays was counterbalanced (81 trials each). Trials were self-terminated and written feedback was presented for half a second after registering the response of the participants (their judgements about the number of targets). Ten practice trials were completed at the beginning of the counting task. The experiment lasted approximately 45 min.

## Results

Data from five participants were removed because of unsuccessful mood manipulation. Three participants’ data was excluded from analysis because of error rates exceeding 50% (71.8%, 77.78%, 90.12%). Total number of participants in each group was nine persons. Data from error trials (8.71%) were excluded from further analysis. Reaction times falling outside two and a half standard deviations (2.5 *SD*) from each participant’s mean reaction time were removed for each of the positive and negative face displays to control for outliers.

### Mood manipulation check

In all groups, participants’ mood score before the mood induction had a slight tendency towards happy mood. Analysis of the mood scores showed that in the short-lived happy mood induction group, participants reported a more pleasant mood after mood manipulation (*M* = 5.56, *SD* = .53) compared with before mood manipulation (*M* = 4.89, *SD* = .78). This means that happy mood induction significantly changed the participant mood, *t*(8) = 2.31, *p* = .05. The difference between scores before (*M* = 5.56, *SD* = .73) and after (*M* = 3.77, *SD* = .97) short-lived sad mood manipulation was significant, *t*(8) = 6.4, *p* < .001. In the prolonged mood manipulation condition, there was a significant difference between mood scores before (*M* = 5.00, *SD* = 1.12) and after (*M* = 5.56, *SD* = 1.13) listening to happy music and recalling happy memory, *t*(8) = 2.29, *p* = .05. Participants in the prolonged sad mood induction group reported a less pleasant mood after listening to sad music and remembering sad memory (*M* = 3.78, *SD* = 1.39) compared to before mood induction (*M* = 5.33, *SD* = 1.00), *t*(8) = 5.29, *p* = .001. This means that in all groups mood manipulation was successful.

### Reaction time analysis

Analysis of reaction time data for different face displays indicated that reaction time was significantly faster, *F*(1, 32) = 15.64, *p* <.001, η^2^ = .33, in response to positive face displays (*M* = 2422 ms, *SD* = 405) than to negative face displays (*M* = 2496 ms, *SD* = 432). That is, attending to the local features of the sad faces is 75 ms slower than attending to the elemental parts of the happy faces.

The main effects of mood (*p* = .08) and MIP (*p* = .14) were not significant.

The interaction between mood and MIP was significant, *F*(1, 32) = 6.89, *p* < .01, η^2^ = .18. Post hoc comparison using Least Significant Difference (LSD) showed that in the prolonged mood induction condition, local processing is significantly slower in happy compared to sad mood, *p* = .004; but the reaction time in short-lived happy and sad mood was not different, *p* = .57. The interaction between face display, mood, and MIP was also significant, *F*(1, 32) = 5.5, *p* < .02, η^2^ = .15. Post hoc comparison using LSD showed that the reaction time to the happy face display in prolonged happy mood was 465 ms slower than reaction time in short-lived happy mood, *p* = .01. Similarly, the reaction time to sad face displays was also 547 ms slower in prolonged compared to short-lived happy mood, *p* = .005 .

**Figure 2.**
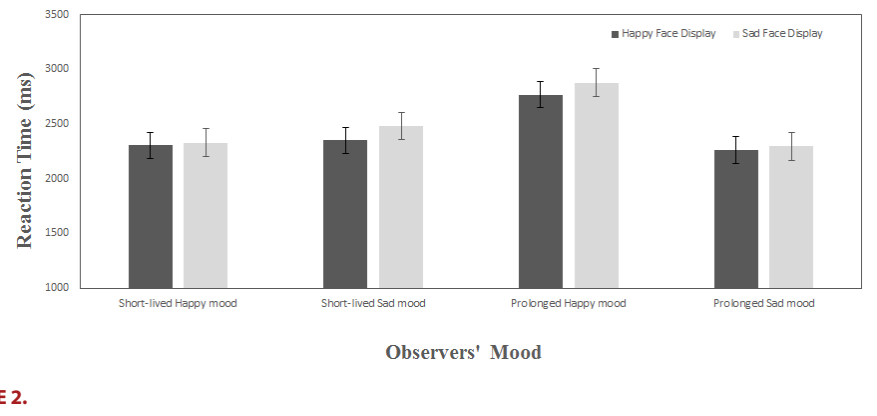
Mean reaction time values (ms) representing speed of detecting and counting a target shape embedded in positive and negative face displays in short-lived and prolonged happy or sad mood. Counting the elemental parts of the negative face displays was slower than positive face displays. Counting parts of both face displays were slower in prolonged happy mood compared to the rest of the mood conditions. Reaction time difference in short-lived happy mood, short-lived sad mood and prolonged sad mood was not significant. Error bars represent ±1 standard error of the mean.

The interaction between mood and face display, *p* = .7, and the interaction between MIP and face display, *p* = .85, were not significant. Figure 2 shows the mean reaction time for mood induction groups and face displays.

### Response accuracy analysis

**Figure 3.**
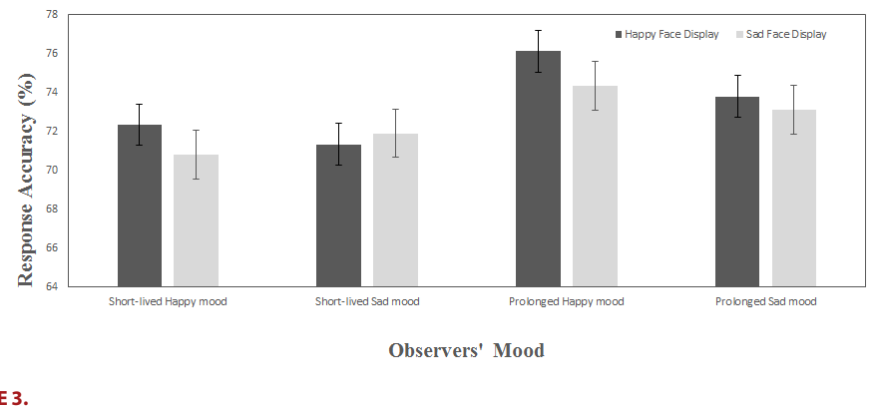
Mean response accuracy (%) representing percentage of correct detection and counting a target shape embedded in positive and negative face displays in short-lived and prolonged happy or sad mood. Compared to the short-lived mood states, the percentage of accurate responses was significantly higher in prolonged mood states. The rest of the main effects and interactions were not significant. Error bars represent ±1 standard error of the mean.

The main effect of MIP was significant, *F*(1, 32) = 6.19, *p* < .01, η^2^ = .18. That is, percentages of correct responses in prolonged mood (*M* = 74.33, *SD* = 3.06) were significantly higher than percentages of correct responses in short-lived mood (*M* = 71.58, *SD* = 3.9). No significant main effect of the face displays was detected, *F*(1, 32) = 2.47, *p* = .13, η^2^ = .07. The main effect of mood valence was not significant, *F*(1, 32) = .677, *p* = .42, η^2^ = .02. Moreover, none of the interactions were significant. Figure 3 shows the mean response accuracy for mood induction group and face displays.

## Discussion

In the present experiment we studied the effect of happy and sad affective states—which varied in persistence—on the processing of the elemental parts of emotional faces. Our task demands attending to the local features of emotional faces. This required overcoming the emotional information conveyed by the prominent global level of the faces and adopting a local processing strategy. The results showed the local perceptual strategy required by the instruction is influenced by the emotional content of the global level stimulus (i.e., the faces’ affective expressions), affective states of observers, and also the duration of experiencing an affective state.

The emotional information conveyed by the global level of the face has an impact on the efficiency of attending to the local features. We observed that, compared with positive faces, negative expressive faces interfered with fast access to the local details. Consistent with the results of Eastwood et al., we propose that negative facial expression, compared to positive, “involuntarily attracts” (Eastwood et al., 2003, p.358) and engages attentional resources (White, 1996) for a longer time. This interferes with efficient attentional switching to the local details. This happens even when the emotional valence of the overall face gestalt is irrelevant for the task. Presumably, the priority of negative facial expressions in directing attention has evolved during the course of time to protect humans from potential dangers in the environment (Öhman & Mineka, 2001).

Our findings also suggested that affective states of an observer have an impact on the level of visual processing; but, that this effect is a function of duration of involvement with the mood. We did not observe any significant difference between sad and happy short-lived moods. One possible explanation is that engaging with the task distracts participants from their mood when it was temporarily induced before that task. For successful completion of the task, some cognitive representations need to be built up or activated; through this, mood-related thoughts were maybe replaced by task related representations (Erber & Tesser, 1992; Morrow & Nolen-Hoeksema, 1990). Van Dillen and Koole (2007, 2011) suggested that processing capacity of working memory is limited and when it is engaged by the task-relevant information, there will not be enough space for mood-related materials.

However, when manipulation of observers’ mood was continued during the course of the task in the form of background music, the likelihood of ignoring the mood state was minimized and existing mood state was used as a source of information to evaluate the available mental resources. In line with the level-of-focus hypothesis (Gasper & Clore, 2002) we observed that prolonged happy mood, compared with prolonged sad mood, delayed the reaction time and interfered with attending to the local features. Our target detection task required efficient suppression of global processing and switching of attention to the local elements. Similar to the assumption of the level-of-focus hypothesis, we found happy individuals had more difficulty in adopting a local processing strategy. However, our results imply that happy mood can be used as information about the value of accessible cognitive strategies if it is available simultaneously with the task.

Rowe, Hirsh and Anderson (2007) suggested that the ability of suppressing the irrelevant information is weakened in happy mood and as a result individuals in happy mood are more distracted by irrelevant information presented at the global level. Phillips, Bull, Adams, and Fraser (2002) observed that individuals in happy mood have impaired performance in a Stroop task which required effortful switching of attention. They interpreted that diffusion of available mental resources in happy mood makes the suppression of the unwanted resources and switch to the most relevant strategy harder for the observer (Phillips et al., 2002). However, our results suggest that happy mood can create diffusion in thoughts and mental resources if it is experienced for a longer duration.

It is important to note that it may seem that faster tempo of happy background music has interfered with completing the task and increased the reaction time. However, previous studies have not supported any impact of background music tempo on cognitive performances (Kampfe, Sedlmeier, & Renkewitz, 2010). In a meta-analysis of 16 studies Kampfe et al. showed that the fast tempo of the music had no overall effect on cognitive performance. Thompson, Schellenberg, and Letnic (2011) showed that fast paced music interferes with cognitive performance only when it is presented at loud volume (72.5 dB); while, the background music in this experiment was presented in medium volume (around 65 dB) as if a radio had been playing in the background. Day, Lin, Huang, and Chuang (2009) also showed that fast music tempo had no effect on the reaction time, although it promotes accurate responses. Taken together, we would argue that the slower reaction time observed in happy mood is less likely to be the effect of fast music tempo but most probably is the effect of prolonged happy mood.

Our results did not show any significant difference between prolonged and short-lived sad mood. The nature of the target detection task might be the reason for this null difference: In most of the previous studies, participants have been given a visual matching task in which the global level of the target can be matched with one of the alternatives and target’s local details can be paired with another alternative (e.g., Gasper, 2004; Gasper & Clore, 2002); however, a target detection task requires active attentional control in which the irrelevant information should be suppressed and task-appropriate perceptual and attentional strategies should be adopted (Bauman & Kuhl, 2005). Previous research showed that laboratory-induced sad mood has no effect on attentional control (e.g., Chepenik et al., 2007). It is also possible that the longevity of sad mood is not a defining factor in the relationship of mood, attention and perception.

Our results did not show any interaction between participants’ mood and stimuli’s emotional meaning (i.e., positive or negative facial expressions). This means that observers’ short-lived or prolonged affective states did not result in mood-congruent stimuli preferences. Vrijsen, Oostrom, Speckens, Becker, and Rinck (2013) discussed that mood-congruent cognitive biases have been mostly observed in clinical depression and laboratory-induced moods do not necessarily lead to mood-congruent stimuli preferences.

### Limitations and the direction of future research

Our study provides stimulating results for researchers who are interested in studying the effect of mood on attention and perception. However, the current research is still preliminary in many aspects. First, in this research we only checked participants’ mood before and after mood induction procedures. However, to assure that response patterns are the function of induced mood, participants’ mood should be ideally measured repeatedly during the course of the experiment.

Future work on developing efficient techniques for prolonged manipulation of participants’ mood in laboratory settings is required. Currently background music is the most popular mood induction technique to sustain participants’ mood during the experimental sessions. However, a couple of factors mitigate the suitability of background music as a mood induction technique: First, effectiveness of the music mood induction procedure considerably depends on people’s individual preferences (Västfjäll, 2002). These individual differences may play the role of a mediating factor in the relation of mood and cognitive performance. Second, in a recent study, Bottiroli, Rosi, Russo, Vecchi, and Cavalini (2014) observed that background music, regardless of its emotional valence, improves older adults’ performance in memory tasks. Similar to their results, we observed improvement in response accuracy in both happy and sad prolonged (background music) mood groups. Although the nature of the task and the participants’ age range in our study were different than Bottiroli et al. (2014), it posits that the effect of background music on cognitive function may be separate from its emotional impacts. Last, the effect of background music does not remain stable during the course of a task. In a study, Eich and Metcalfe (1989) employed background music to induce happy and sad mood in their participants. To check the manipulation success, they measured participants’ current mood every five minutes. Data revealed that background music had the highest influence on participants’ mood at the beginning and at the end of the experimental session. This means that towards the middle part of the task participants rated their mood less happy (in case of listening to a happy music) or less sad (in case of listening to a sad music). This “affective adaptation” (Västfjäll, 2002, p.193) might have an impact on cognitive performance.

The default perceptual strategy and its effect on the relation of mood and cognition should also be attended by future research. Recent studies suggest that the impact of mood on perception is “malleable” and depends on the default perceptual strategy (Hunsinger, Isbell, & Clore, 2012; Huntsinger, 2013; Huntsinger, Clore, & Bar-Anan, 2010). This hypothesis implies that happy mood promotes any adopted perceptual strategy, while sad mood inhibits it. Since global processing is the common strategy in visual perception (Kimchi, 1992; Navon, 1977), happy mood mostly tends to facilitate global processing and sad mood mostly promotes local processing. However, if the observer’s default processing strategy is primed to local processing, happy mood promotes attending to those local details (Hunsinger et al., 2012; Huntsinger, 2013; Huntsinger et al., 2010).

### Conclusion

Our findings suggest that the interrelation between mood, attention and perception is complex and for the better understanding of this relationship other items like the duration of experiencing the mood, the mood induction techniques and the task demands should be taken to account.
